# 4-[2-(Anthracen-9-yl­methyl­idene)hydrazinyl­idene]-3-chloro-1-methyl-3,4-dihydro-1*H*-2λ^6^,1-benzothia­zine-2,2-dione

**DOI:** 10.1107/S1600536812021642

**Published:** 2012-05-19

**Authors:** Muhammad Shafiq, M. Nawaz Tahir, Islam Ullah Khan, Tanveer Hussain Bokhari, Muhammad Nadeem Asghar

**Affiliations:** a Department of Chemistry, Government College University, Faisalabad, Pakistan; b University of Sargodha, Department of Physics, Sargodha, Pakistan; c Materials Chemistry Laboratory, Department of Chemistry, Government College University, Lahore, Pakistan; d Forman Christian College (A Chartered University), Ferozepur Road, Lahore-54600, Pakistan

## Abstract

In the title compound, C_24_H_18_ClN_3_O_2_S, the dihedral angle between the benzene ring and the anthracene ring system is 41.10 (8)°. The thia­zine ring has a half-chair conformation and the Cl atom is in an axial orientation. In the crystal, mol­ecules are linked by C—H⋯O inter­actions, generating *C*(8) chains along [100]. A C—H⋯N short contact occurs in the mol­ecule, generating an *S*(6) ring.

## Related literature

For a related structure and references to further synthetic details, see: Shafiq *et al.* (2012 ▶). For puckering parameters, see: Cremer & Pople (1975 ▶). For hydrogen-bond motifs, see: Bernstein *et al.* (1995 ▶).
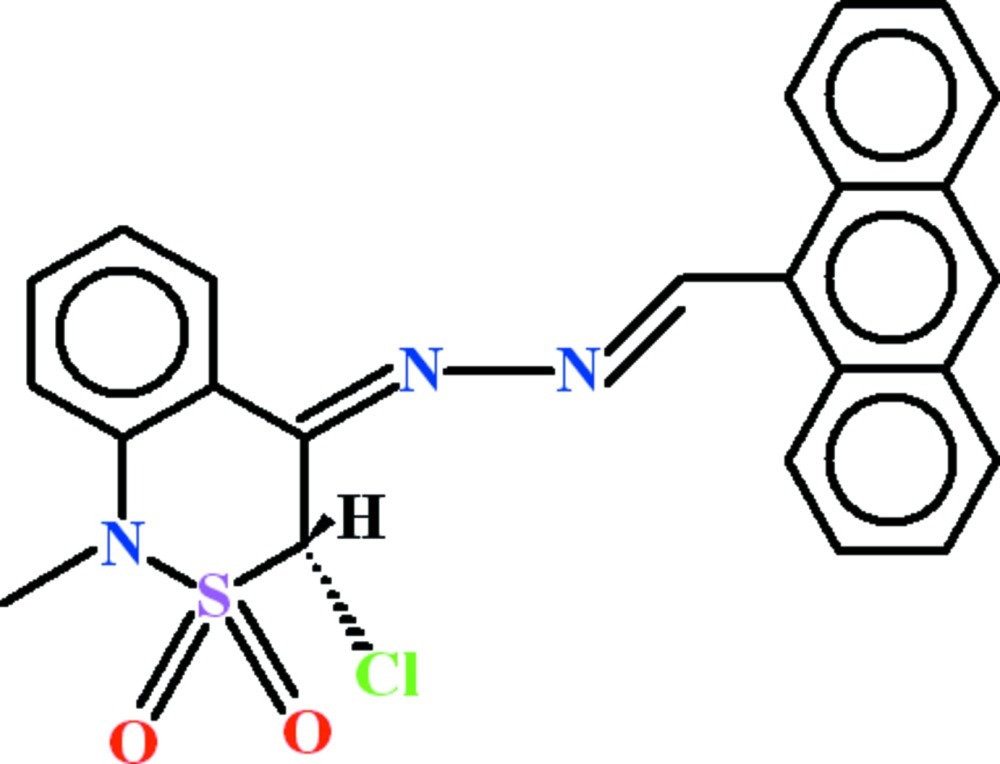


## Experimental

### 

#### Crystal data

C_24_H_18_ClN_3_O_2_S
*M* *_r_* = 447.92
Monoclinic, 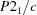

*a* = 8.5133 (4) Å
*b* = 19.8999 (8) Å
*c* = 12.7849 (6) Å
β = 105.026 (2)°
*V* = 2091.88 (16) Å^3^
*Z* = 4
Mo *K*α radiation
μ = 0.31 mm^−1^
*T* = 296 K
0.26 × 0.22 × 0.20 mm

#### Data collection

Bruker Kappa APEXII CCD diffractometer
Absorption correction: multi-scan (*SADABS*; Bruker, 2005 ▶) *T*_min_ = 0.930, *T*_max_ = 0.960
16154 measured reflections
3788 independent reflections
2827 reflections with *I* > 2σ(*I*)
*R*_int_ = 0.031

#### Refinement

*R*[*F* ^2^ > 2σ(*F* ^2^)] = 0.047
*wR*(*F* ^2^) = 0.136
*S* = 1.04
3788 reflections
281 parameters
H-atom parameters constrained
Δρ_max_ = 0.47 e Å^−3^
Δρ_min_ = −0.35 e Å^−3^

### 

Data collection: *APEX2* (Bruker, 2009 ▶); cell refinement: *SAINT* (Bruker, 2009 ▶); data reduction: *SAINT*; program(s) used to solve structure: *SHELXS97* (Sheldrick, 2008 ▶); program(s) used to refine structure: *SHELXL97* (Sheldrick, 2008 ▶); molecular graphics: *ORTEP-3 for Windows* (Farrugia, 1997 ▶) and *PLATON* (Spek, 2009 ▶); software used to prepare material for publication: *WinGX* (Farrugia, 1999 ▶) and *PLATON*.

## Supplementary Material

Crystal structure: contains datablock(s) global, I. DOI: 10.1107/S1600536812021642/hb6779sup1.cif

Structure factors: contains datablock(s) I. DOI: 10.1107/S1600536812021642/hb6779Isup2.hkl

Supplementary material file. DOI: 10.1107/S1600536812021642/hb6779Isup3.cml

Additional supplementary materials:  crystallographic information; 3D view; checkCIF report

## Figures and Tables

**Table 1:** Hydrogen-bond geometry (Å, °)

*D*—H⋯*A*	*D*—H	H⋯*A*	*D*⋯*A*	*D*—H⋯*A*
C3—H3⋯O2^i^	0.93	2.59	3.370 (4)	142
C22—H22⋯N3	0.93	2.29	2.913 (4)	123

Symmetry code: (i) 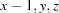.

**Fig. 1.**
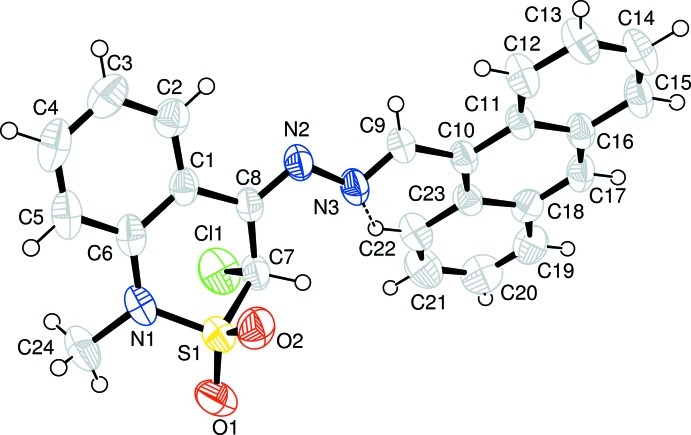
View of the title compound with displacement ellipsoids drawn at the 50% probability level. The dotted line indicates the intra-molecular short contact.

**Fig. 2.**
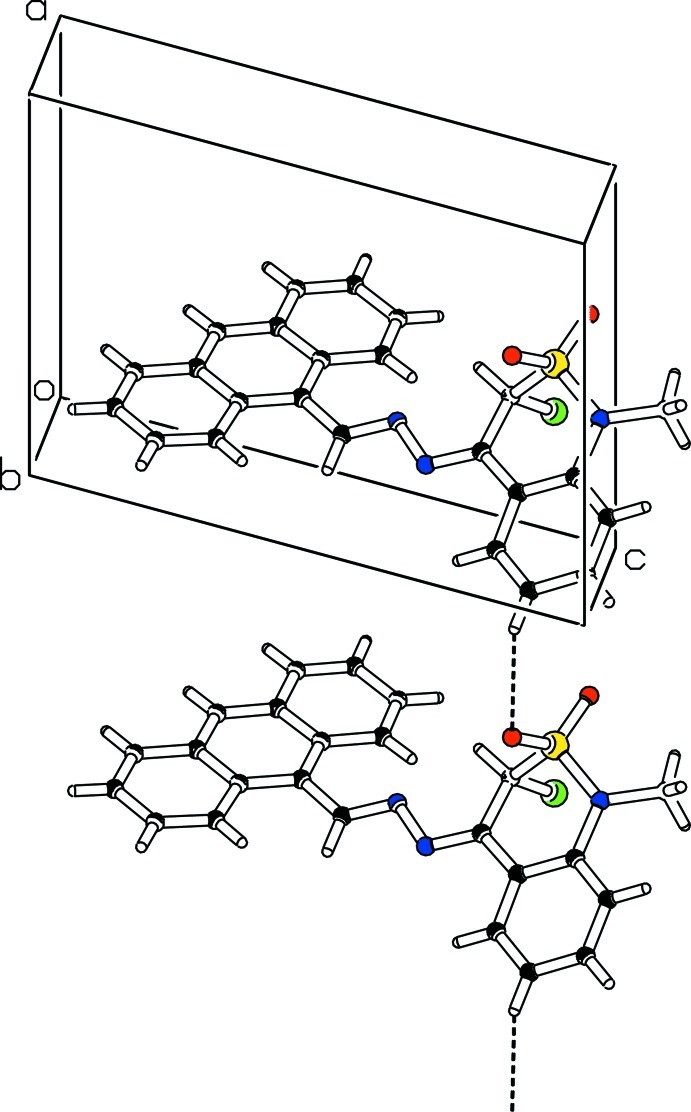
The partial packing, which shows that molecules form one-dimensional polymeric chains extending along the [100] direction.

## supplementary crystallographic information

### Comment

As part of our ongoing studies of thiazine derivatives (Shafiq *et al.*, 2012), we now describe the structure of the title compound, (I), (Fig. 1).

In (I), the benzene ring A (C1—C6) and anthracene group B (C10—C23) are almost planar with r. m. s. deviation of 0.0090 and 0.0144 Å, respectively. The dihedral angle between A/B is 41.10 (8)°. The central group C (N2/N3/C9) is of course planar. The dihedral angle between A/C and B/C is 13.63 (26) and 27.48 (26)°, respectively. The thiazine ring D (C1/C6/C7/C8/S1/N1) is in the half-chair form, with the maximum puckering amplitude (Cremer & Pople, 1975), Q = 0.578 (2) Å. There exist *S*(6) ring motif (Bernstein *et al.*, 1995) due to H-bonding of C—H···N type (Table 1, Fig. 1). The molecules form C(8) chains extending along the *a*-axis due to H-bonding of C—H···O type (Table 1, Fig. 2).

### Experimental

Schiff base derivative of (4*Z*)-4-hydrazinylidene-1-methyl-3,4-dihydro -1*H*-2,1-benzothiazine 2,2-dioxide and anthracene-9-carbaldehyde was prepared using the method reported previously (Shafiq *et al.* 2012). The chlorination of the schiff base was undertaken using *N*-chloro succinimide and dibenzoylperoxide (Shafiq *et al.*, 2012). The crude product of (I) was re-crystallized in ethyl acetate to get orange prisms.

### Refinement

The H-atoms were positioned geometrically (C–H = 0.93–0.98 Å) and refined as riding with *U*_iso_(H) = *xU*_eq_(C), where *x* = 1.5 for methyl and *x* = 1.2 for aryl H-atoms.

### Crystal data

**Table:**

C_24_H_18_ClN_3_O_2_S	*F*(000) = 928
*M**_r_* = 447.92	*D*_x_ = 1.422 Mg m^−^^3^
Monoclinic, *P*2_1_/*c*	Mo *K*α radiation, λ = 0.71073 Å
Hall symbol: -P 2ybc	Cell parameters from 2827 reflections
*a* = 8.5133 (4) Å	θ = 2.1–25.3°
*b* = 19.8999 (8) Å	µ = 0.31 mm^−^^1^
*c* = 12.7849 (6) Å	*T* = 296 K
β = 105.026 (2)°	Prism, orange
*V* = 2091.88 (16) Å^3^	0.26 × 0.22 × 0.20 mm
*Z* = 4	

### Data collection

**Table:**

Bruker Kappa APEXII CCD diffractometer	3788 independent reflections
Radiation source: fine-focus sealed tube	2827 reflections with *I* > 2σ(*I*)
Graphite monochromator	*R*_int_ = 0.031
Detector resolution: 8.10 pixels mm^-1^	θ_max_ = 25.3°, θ_min_ = 2.1°
ω scans	*h* = −10→9
Absorption correction: multi-scan (*SADABS*; Bruker, 2005)	*k* = −23→18
*T*_min_ = 0.930, *T*_max_ = 0.960	*l* = −15→15
16154 measured reflections	

### Refinement

**Table:**

Refinement on *F*^2^	Primary atom site location: structure-invariant direct methods
Least-squares matrix: full	Secondary atom site location: difference Fourier map
*R*[*F*^2^ > 2σ(*F*^2^)] = 0.047	Hydrogen site location: inferred from neighbouring sites
*wR*(*F*^2^) = 0.136	H-atom parameters constrained
*S* = 1.04	*w* = 1/[σ^2^(*F*_o_^2^) + (0.0652*P*)^2^ + 0.8876*P*] where *P* = (*F*_o_^2^ + 2*F*_c_^2^)/3
3788 reflections	(Δ/σ)_max_ = 0.001
281 parameters	Δρ_max_ = 0.47 e Å^−^^3^
0 restraints	Δρ_min_ = −0.35 e Å^−^^3^

### Special details

**Table:**

Geometry. Bond distances, angles *etc*. have been calculated using the rounded fractional coordinates. All su's are estimated from the variances of the (full) variance-covariance matrix. The cell e.s.d.'s are taken into account in the estimation of distances, angles and torsion angles
Refinement. Refinement of *F*^2^ against ALL reflections. The weighted *R*-factor *wR* and goodness of fit *S* are based on *F*^2^, conventional *R*-factors *R* are based on *F*, with *F* set to zero for negative *F*^2^. The threshold expression of *F*^2^ > σ(*F*^2^) is used only for calculating *R*-factors(gt) *etc*. and is not relevant to the choice of reflections for refinement. *R*-factors based on *F*^2^ are statistically about twice as large as those based on *F*, and *R*- factors based on ALL data will be even larger.

### Fractional atomic coordinates and isotropic or equivalent isotropic displacement parameters (Å^2^)

**Table:**

	*x*	*y*	*z*	*U*_iso_*/*U*_eq_	
Cl1	0.31773 (12)	0.02405 (4)	0.89027 (7)	0.0772 (3)	
S1	0.47338 (9)	0.15552 (4)	0.89884 (6)	0.0561 (3)	
O1	0.6215 (3)	0.12587 (13)	0.95945 (17)	0.0790 (8)	
O2	0.4770 (2)	0.20988 (10)	0.82508 (16)	0.0625 (7)	
N1	0.3657 (3)	0.17603 (12)	0.98246 (17)	0.0581 (8)	
N2	0.1106 (3)	0.12240 (10)	0.66521 (16)	0.0470 (7)	
N3	0.1929 (3)	0.07899 (11)	0.61082 (16)	0.0520 (8)	
C1	0.1136 (3)	0.17431 (11)	0.8339 (2)	0.0436 (8)	
C2	−0.0466 (3)	0.19551 (13)	0.7960 (2)	0.0533 (9)	
C3	−0.1214 (4)	0.23414 (14)	0.8580 (3)	0.0632 (11)	
C4	−0.0340 (4)	0.25376 (14)	0.9606 (3)	0.0665 (13)	
C5	0.1253 (4)	0.23567 (14)	0.9993 (2)	0.0596 (10)	
C6	0.2010 (4)	0.19540 (13)	0.9382 (2)	0.0498 (9)	
C7	0.3445 (3)	0.09596 (13)	0.8164 (2)	0.0485 (8)	
C8	0.1851 (3)	0.13033 (12)	0.76582 (18)	0.0414 (8)	
C9	0.1181 (3)	0.07213 (12)	0.51179 (19)	0.0456 (8)	
C10	0.1765 (3)	0.03012 (12)	0.43534 (19)	0.0430 (8)	
C11	0.1245 (3)	0.04805 (12)	0.32402 (19)	0.0456 (8)	
C12	0.0261 (4)	0.10529 (14)	0.2848 (2)	0.0619 (10)	
C13	−0.0196 (4)	0.12103 (16)	0.1775 (2)	0.0676 (10)	
C14	0.0306 (4)	0.08209 (16)	0.1014 (2)	0.0667 (10)	
C15	0.1244 (4)	0.02818 (15)	0.1340 (2)	0.0603 (10)	
C16	0.1755 (3)	0.00887 (13)	0.2453 (2)	0.0473 (8)	
C17	0.2714 (3)	−0.04692 (13)	0.2787 (2)	0.0534 (10)	
C18	0.3237 (3)	−0.06616 (12)	0.3865 (2)	0.0490 (9)	
C19	0.4221 (4)	−0.12438 (15)	0.4175 (3)	0.0641 (11)	
C20	0.4705 (4)	−0.14389 (16)	0.5208 (3)	0.0742 (13)	
C21	0.4206 (4)	−0.10726 (15)	0.6017 (3)	0.0723 (11)	
C22	0.3269 (4)	−0.05134 (14)	0.5762 (2)	0.0596 (10)	
C23	0.2760 (3)	−0.02744 (12)	0.4675 (2)	0.0447 (8)	
C24	0.4352 (5)	0.1704 (2)	1.0998 (2)	0.0851 (15)	
H2	−0.10484	0.18318	0.72662	0.0639*	
H3	−0.22948	0.24698	0.83144	0.0757*	
H4	−0.08417	0.27945	1.00339	0.0798*	
H5	0.18372	0.25048	1.06725	0.0716*	
H7	0.39378	0.08215	0.75851	0.0582*	
H9	0.02056	0.09509	0.48613	0.0547*	
H12	−0.00791	0.13259	0.33383	0.0744*	
H13	−0.08517	0.15836	0.15457	0.0813*	
H14	−0.00106	0.09351	0.02836	0.0801*	
H15	0.15741	0.00253	0.08271	0.0725*	
H17	0.30231	−0.07276	0.22679	0.0640*	
H19	0.45336	−0.14929	0.36469	0.0764*	
H20	0.53679	−0.18145	0.53962	0.0887*	
H21	0.45241	−0.12174	0.67323	0.0865*	
H22	0.29546	−0.02823	0.63070	0.0713*	
H24A	0.46719	0.21406	1.12959	0.1277*	
H24B	0.52845	0.14138	1.11401	0.1277*	
H24C	0.35525	0.15194	1.13268	0.1277*	

### Atomic displacement parameters (Å^2^)

**Table:**

	*U*^11^	*U*^22^	*U*^33^	*U*^12^	*U*^13^	*U*^23^
Cl1	0.1028 (7)	0.0585 (5)	0.0677 (5)	0.0011 (4)	0.0176 (5)	0.0132 (4)
S1	0.0554 (4)	0.0686 (5)	0.0428 (4)	−0.0061 (3)	0.0100 (3)	−0.0092 (3)
O1	0.0610 (13)	0.1054 (17)	0.0592 (13)	0.0087 (12)	−0.0049 (10)	−0.0130 (12)
O2	0.0646 (13)	0.0666 (12)	0.0617 (12)	−0.0183 (10)	0.0263 (10)	−0.0031 (10)
N1	0.0694 (16)	0.0683 (15)	0.0333 (12)	−0.0026 (12)	0.0076 (11)	−0.0079 (11)
N2	0.0551 (13)	0.0515 (12)	0.0352 (11)	0.0019 (10)	0.0131 (10)	−0.0046 (9)
N3	0.0600 (14)	0.0615 (13)	0.0347 (12)	0.0041 (11)	0.0127 (10)	−0.0103 (10)
C1	0.0547 (16)	0.0401 (13)	0.0410 (14)	−0.0090 (11)	0.0215 (12)	−0.0026 (11)
C2	0.0561 (17)	0.0506 (15)	0.0574 (17)	−0.0084 (13)	0.0225 (14)	−0.0053 (13)
C3	0.0625 (19)	0.0526 (16)	0.085 (2)	−0.0038 (14)	0.0378 (17)	−0.0041 (16)
C4	0.095 (3)	0.0477 (16)	0.075 (2)	−0.0053 (16)	0.055 (2)	−0.0096 (15)
C5	0.091 (2)	0.0487 (16)	0.0480 (16)	−0.0079 (15)	0.0339 (16)	−0.0090 (13)
C6	0.0706 (19)	0.0436 (14)	0.0406 (14)	−0.0100 (13)	0.0239 (13)	−0.0041 (11)
C7	0.0562 (16)	0.0547 (15)	0.0354 (13)	−0.0003 (12)	0.0132 (12)	−0.0057 (11)
C8	0.0504 (15)	0.0433 (13)	0.0330 (13)	−0.0080 (11)	0.0152 (11)	−0.0037 (10)
C9	0.0561 (16)	0.0464 (14)	0.0343 (13)	−0.0019 (12)	0.0120 (12)	−0.0022 (11)
C10	0.0514 (15)	0.0441 (13)	0.0337 (13)	−0.0074 (11)	0.0114 (11)	−0.0054 (10)
C11	0.0569 (16)	0.0455 (14)	0.0335 (13)	−0.0095 (12)	0.0103 (11)	−0.0059 (11)
C12	0.089 (2)	0.0559 (16)	0.0392 (15)	0.0073 (15)	0.0136 (14)	−0.0011 (13)
C13	0.094 (2)	0.0603 (17)	0.0434 (16)	0.0009 (16)	0.0084 (16)	0.0050 (14)
C14	0.096 (2)	0.0651 (19)	0.0341 (15)	−0.0196 (18)	0.0083 (15)	0.0018 (14)
C15	0.083 (2)	0.0653 (18)	0.0354 (14)	−0.0200 (16)	0.0202 (14)	−0.0123 (13)
C16	0.0590 (16)	0.0473 (14)	0.0371 (13)	−0.0133 (12)	0.0154 (12)	−0.0083 (11)
C17	0.0653 (18)	0.0554 (16)	0.0461 (16)	−0.0120 (14)	0.0265 (13)	−0.0138 (13)
C18	0.0510 (16)	0.0436 (14)	0.0555 (17)	−0.0076 (12)	0.0192 (13)	−0.0077 (12)
C19	0.072 (2)	0.0532 (16)	0.073 (2)	0.0051 (15)	0.0296 (17)	−0.0012 (15)
C20	0.078 (2)	0.0541 (18)	0.090 (3)	0.0105 (16)	0.0211 (19)	0.0066 (17)
C21	0.089 (2)	0.0621 (19)	0.0595 (19)	0.0060 (17)	0.0081 (17)	0.0120 (15)
C22	0.077 (2)	0.0533 (16)	0.0454 (16)	0.0013 (14)	0.0103 (14)	0.0003 (13)
C23	0.0504 (15)	0.0436 (13)	0.0386 (13)	−0.0086 (11)	0.0086 (11)	−0.0028 (11)
C24	0.092 (3)	0.124 (3)	0.0344 (16)	−0.008 (2)	0.0078 (16)	−0.0069 (18)

### Geometric parameters (Å, º)

**Table:**

Cl1—C7	1.762 (3)	C16—C17	1.379 (4)
S1—O1	1.426 (3)	C17—C18	1.388 (3)
S1—O2	1.441 (2)	C18—C19	1.424 (4)
S1—N1	1.630 (3)	C18—C23	1.432 (4)
S1—C7	1.766 (3)	C19—C20	1.335 (5)
N1—C6	1.423 (4)	C20—C21	1.418 (5)
N1—C24	1.467 (3)	C21—C22	1.359 (4)
N2—N3	1.405 (3)	C22—C23	1.426 (4)
N2—C8	1.288 (3)	C2—H2	0.9300
N3—C9	1.269 (3)	C3—H3	0.9300
C1—C2	1.389 (4)	C4—H4	0.9300
C1—C6	1.412 (4)	C5—H5	0.9300
C1—C8	1.473 (3)	C7—H7	0.9800
C2—C3	1.374 (4)	C9—H9	0.9300
C3—C4	1.386 (5)	C12—H12	0.9300
C4—C5	1.366 (5)	C13—H13	0.9300
C5—C6	1.389 (4)	C14—H14	0.9300
C7—C8	1.508 (4)	C15—H15	0.9300
C9—C10	1.467 (3)	C17—H17	0.9300
C10—C11	1.422 (3)	C19—H19	0.9300
C10—C23	1.421 (3)	C20—H20	0.9300
C11—C12	1.426 (4)	C21—H21	0.9300
C11—C16	1.427 (4)	C22—H22	0.9300
C12—C13	1.362 (4)	C24—H24A	0.9600
C13—C14	1.395 (4)	C24—H24B	0.9600
C14—C15	1.338 (4)	C24—H24C	0.9600
C15—C16	1.429 (4)		
			
O1—S1—O2	120.04 (14)	C18—C19—C20	121.3 (3)
O1—S1—N1	108.40 (13)	C19—C20—C21	119.9 (3)
O1—S1—C7	111.82 (14)	C20—C21—C22	121.0 (3)
O2—S1—N1	110.63 (12)	C21—C22—C23	121.2 (3)
O2—S1—C7	103.22 (12)	C10—C23—C18	119.0 (2)
N1—S1—C7	101.03 (13)	C10—C23—C22	124.0 (2)
S1—N1—C6	118.09 (17)	C18—C23—C22	117.0 (2)
S1—N1—C24	120.3 (2)	C1—C2—H2	119.00
C6—N1—C24	121.5 (3)	C3—C2—H2	119.00
N3—N2—C8	113.0 (2)	C2—C3—H3	120.00
N2—N3—C9	112.0 (2)	C4—C3—H3	120.00
C2—C1—C6	117.9 (2)	C3—C4—H4	120.00
C2—C1—C8	119.6 (2)	C5—C4—H4	120.00
C6—C1—C8	122.5 (2)	C4—C5—H5	120.00
C1—C2—C3	121.9 (3)	C6—C5—H5	120.00
C2—C3—C4	119.2 (3)	Cl1—C7—H7	109.00
C3—C4—C5	120.5 (3)	S1—C7—H7	109.00
C4—C5—C6	120.7 (3)	C8—C7—H7	108.00
N1—C6—C1	121.3 (2)	N3—C9—H9	118.00
N1—C6—C5	119.1 (2)	C10—C9—H9	118.00
C1—C6—C5	119.7 (3)	C11—C12—H12	119.00
Cl1—C7—S1	111.83 (14)	C13—C12—H12	119.00
Cl1—C7—C8	111.80 (18)	C12—C13—H13	120.00
S1—C7—C8	107.60 (17)	C14—C13—H13	119.00
N2—C8—C1	119.4 (2)	C13—C14—H14	120.00
N2—C8—C7	121.9 (2)	C15—C14—H14	120.00
C1—C8—C7	118.7 (2)	C14—C15—H15	119.00
N3—C9—C10	123.9 (2)	C16—C15—H15	119.00
C9—C10—C11	116.9 (2)	C16—C17—H17	119.00
C9—C10—C23	123.1 (2)	C18—C17—H17	119.00
C11—C10—C23	120.0 (2)	C18—C19—H19	119.00
C10—C11—C12	123.7 (2)	C20—C19—H19	119.00
C10—C11—C16	119.7 (2)	C19—C20—H20	120.00
C12—C11—C16	116.6 (2)	C21—C20—H20	120.00
C11—C12—C13	121.8 (3)	C20—C21—H21	119.00
C12—C13—C14	121.1 (3)	C22—C21—H21	120.00
C13—C14—C15	119.6 (2)	C21—C22—H22	119.00
C14—C15—C16	121.9 (3)	C23—C22—H22	119.00
C11—C16—C15	119.1 (2)	N1—C24—H24A	110.00
C11—C16—C17	119.1 (2)	N1—C24—H24B	110.00
C15—C16—C17	121.9 (2)	N1—C24—H24C	109.00
C16—C17—C18	122.8 (2)	H24A—C24—H24B	110.00
C17—C18—C19	121.0 (3)	H24A—C24—H24C	109.00
C17—C18—C23	119.5 (2)	H24B—C24—H24C	109.00
C19—C18—C23	119.6 (2)		
			
O1—S1—N1—C6	−169.0 (2)	S1—C7—C8—C1	−43.3 (3)
O1—S1—N1—C24	7.3 (3)	N3—C9—C10—C11	−154.0 (3)
O2—S1—N1—C6	57.5 (2)	N3—C9—C10—C23	28.1 (4)
O2—S1—N1—C24	−126.2 (2)	C9—C10—C11—C12	2.7 (4)
C7—S1—N1—C6	−51.3 (2)	C9—C10—C11—C16	−179.4 (2)
C7—S1—N1—C24	125.0 (3)	C23—C10—C11—C12	−179.3 (3)
O1—S1—C7—Cl1	50.62 (19)	C23—C10—C11—C16	−1.3 (4)
O1—S1—C7—C8	173.77 (17)	C9—C10—C23—C18	178.5 (2)
O2—S1—C7—Cl1	−178.99 (14)	C9—C10—C23—C22	1.3 (4)
O2—S1—C7—C8	−55.85 (19)	C11—C10—C23—C18	0.7 (4)
N1—S1—C7—Cl1	−64.50 (17)	C11—C10—C23—C22	−176.6 (3)
N1—S1—C7—C8	58.64 (19)	C10—C11—C12—C13	179.3 (3)
S1—N1—C6—C1	23.8 (3)	C16—C11—C12—C13	1.3 (4)
S1—N1—C6—C5	−156.4 (2)	C10—C11—C16—C15	−179.0 (3)
C24—N1—C6—C1	−152.4 (3)	C10—C11—C16—C17	1.6 (4)
C24—N1—C6—C5	27.4 (4)	C12—C11—C16—C15	−0.9 (4)
C8—N2—N3—C9	−179.6 (2)	C12—C11—C16—C17	179.8 (3)
N3—N2—C8—C1	−179.8 (2)	C11—C12—C13—C14	−1.0 (5)
N3—N2—C8—C7	0.4 (3)	C12—C13—C14—C15	0.4 (5)
N2—N3—C9—C10	179.6 (2)	C13—C14—C15—C16	0.0 (5)
C6—C1—C2—C3	−1.9 (4)	C14—C15—C16—C11	0.3 (5)
C8—C1—C2—C3	177.1 (2)	C14—C15—C16—C17	179.6 (3)
C2—C1—C6—N1	−179.7 (2)	C11—C16—C17—C18	−1.3 (4)
C2—C1—C6—C5	0.5 (4)	C15—C16—C17—C18	179.3 (3)
C8—C1—C6—N1	1.3 (4)	C16—C17—C18—C19	179.7 (3)
C8—C1—C6—C5	−178.6 (2)	C16—C17—C18—C23	0.6 (4)
C2—C1—C8—N2	13.1 (4)	C17—C18—C19—C20	−178.8 (3)
C2—C1—C8—C7	−167.1 (2)	C23—C18—C19—C20	0.3 (5)
C6—C1—C8—N2	−167.9 (2)	C17—C18—C23—C10	−0.3 (4)
C6—C1—C8—C7	11.9 (4)	C17—C18—C23—C22	177.1 (3)
C1—C2—C3—C4	1.3 (4)	C19—C18—C23—C10	−179.4 (3)
C2—C3—C4—C5	0.8 (5)	C19—C18—C23—C22	−2.0 (4)
C3—C4—C5—C6	−2.2 (5)	C18—C19—C20—C21	1.5 (5)
C4—C5—C6—N1	−178.2 (3)	C19—C20—C21—C22	−1.6 (5)
C4—C5—C6—C1	1.6 (4)	C20—C21—C22—C23	−0.2 (5)
Cl1—C7—C8—N2	−100.3 (3)	C21—C22—C23—C10	179.2 (3)
Cl1—C7—C8—C1	79.9 (2)	C21—C22—C23—C18	1.9 (4)
S1—C7—C8—N2	136.5 (2)		

### Hydrogen-bond geometry (Å, º)

**Table:**

*D*—H···*A*	*D*—H	H···*A*	*D*···*A*	*D*—H···*A*
C3—H3···O2^i^	0.93	2.59	3.370 (4)	142
C22—H22···N3	0.93	2.29	2.913 (4)	123

Symmetry code: (i) *x*−1, *y*, *z*.

## References

[bb9] Bernstein, J., Davis, R. E., Shimoni, L. & Chang, N.-L. (1995). *Angew. Chem. Int. Ed. Engl.* **34**, 1555–1573.

[bb1] Bruker (2005). *SADABS* Bruker AXS Inc., Madison, Wisconsin, USA.

[bb2] Bruker (2009). *APEX2* and *SAINT* Bruker AXS Inc., Madison, Wisconsin, USA.

[bb3] Cremer, D. & Pople, J. A. (1975). *J. Am. Chem. Soc.* **97**, 1354–1358.

[bb4] Farrugia, L. J. (1997). *J. Appl. Cryst.* **30**, 565.

[bb5] Farrugia, L. J. (1999). *J. Appl. Cryst.* **32**, 837–838.

[bb6] Shafiq, M., Tahir, M. N., Khan, I. U. & Ahmad, S. (2012). *Acta Cryst.* E**68**, o1787. 10.1107/S1600536812021654 PMC3379366 22719564

[bb7] Sheldrick, G. M. (2008). *Acta Cryst.* A**64**, 112–122. 10.1107/S0108767307043930 18156677

[bb8] Spek, A. L. (2009). *Acta Cryst.* D**65**, 148–155. 10.1107/S090744490804362X PMC2631630 19171970

